# MedPTQ: a practical pipeline for real post-training quantization in 3D medical image segmentation

**DOI:** 10.1117/1.JMI.13.1.014006

**Published:** 2026-02-17

**Authors:** Chongyu Qu, Ritchie Zhao, Ye Yu, Bin Liu, Tianyuan Yao, Junchao Zhu, Bennett A. Landman, Yucheng Tang, Yuankai Huo

**Affiliations:** a Vanderbilt University, Department of Electrical and Computer Engineering, Nashville, Tennessee, United States; b Vanderbilt University, Department of Computer Science, Nashville, Tennessee, United States; c NVIDIA, Santa Clara, California, United States

**Keywords:** quantization, efficiency, medical imaging segmentation

## Abstract

**Purpose:**

Quantizing deep neural networks, reducing the precision (bit-width) of their computations, can remarkably decrease memory usage and accelerate processing, making these models more suitable for large-scale medical imaging applications with limited computational resources. However, many existing methods studied “simulated quantization,” which simulates lower precision operations during inference but does not actually reduce model size or improve real-world inference speed. Moreover, the potential of deploying real three-dimensional (3D) low-bit quantization on modern graphics processing units (GPUs) is still unexplored.

**Approach:**

We introduce MedPTQ, an open-source pipeline for real post-training quantization that implements true 8-bit (INT8) inference on state-of-the-art (SOTA) 3D medical segmentation models, i.e., U-Net, SegResNet, SwinUNETR, nnU-Net, UNesT, TransUNet, ST-UNet, and VISTA3D. MedPTQ involves two main steps. First, we use TensorRT to perform simulated quantization for both weights and activations with an unlabeled calibration dataset. Second, we convert this simulated quantization into real quantization via the TensorRT engine on real GPUs, resulting in real-world reductions in model size and inference latency.

**Results:**

Extensive experiments benchmark MedPTQ across seven models and three datasets and demonstrate that it effectively performs INT8 quantization on GPUs, reducing model size by up to 3.83× and latency by up to 2.74×, while maintaining nearly identical Dice similarity coefficient (mDSC) performance to FP32 models. This advancement enables the deployment of efficient deep learning models in medical imaging applications where computational resources are constrained. The MedPTQ code and models have been released, including U-Net, TransUNet pretrained on the BTCV dataset for abdominal (13-label) segmentation, UNesT pretrained on the Whole Brain Dataset for whole brain (133-label) segmentation, and nnU-Net, SegResNet, SwinUNETR, and VISTA3D pretrained on TotalSegmentator V2 for full body (104-label) segmentation.

**Conclusions:**

We have introduced MedPTQ, a real post-training quantization pipeline that delivers INT8 inference for SOTA 3D artificial intelligence (AI) models in medical imaging segmentation. MedPTQ effectively reduces real-world model size, computational requirements, and inference latency without compromising segmentation accuracy on modern GPUs, as evidenced by mDSC comparable to full-precision baselines. We validate MedPTQ across a diverse set of AI architectures, ranging from convolutional-neural-network-based to transformer-based models, and a wide variety of medical imaging datasets. These datasets are collected from multiple hospitals with distinct imaging protocols, cover different body regions (such as the brain, abdomen, or full body), and include multiple imaging modalities [computed tomography (CT) and magnetic resonance imaging (MRI)]. Collectively, these results highlight our MedPTQ’s strong generalizability and adaptability for a broad spectrum of medical imaging tasks.

## 1 Introduction

Deep neural networks have become indispensable in medical imaging tasks, remarkably enhancing diagnostic accuracy and efficiency in tasks such as image classification,^1^^–^^3^ segmentation,^4^^–^^8^ and anomaly detection.^9^^–^^11^ Despite their effectiveness, deploying these models in large-scale medical imaging applications remains challenging due to substantial computational requirements and limited clinical hardware resources.

A promising approach to mitigate these challenges is model quantization,^12^^–^^15^ which reduces the precision (bit-width) of computations within a neural network. By converting high-precision representations (e.g., FP32) to lower-precision formats (e.g., INT8) for both weights and activations, quantization can remarkably decrease memory usage and accelerate processing speeds. This transformation not only makes models more suitable for deployment on devices with constrained resources but also enables faster inference times essential for real-time medical applications.

Quantization methods are broadly categorized into two types: (1) quantization-aware training (QAT) and (2) post-training quantization (PTQ). QAT trains a model by simulating low-precision calculations during both forward and backward passes, allowing the model to adjust to these constraints. As a result, the model can retain its accuracy after being quantized. For example, DeepSeek V3^16^ introduces an FP8 mixed precision training framework and, for the first time, validates its effectiveness on an extremely large-scale model. By leveraging FP8 computation and storage, DeepSeek V3 achieves both accelerated training and reduced graphics processing unit (GPU) memory usage. Although QAT can achieve high accuracy at lower precisions, it is time-consuming and requires access to the entire labeled training dataset, which is a notable drawback given the massive size and sensitivity of medical imaging data. PTQ quantizes a pre-trained model without the need for retraining, using only a small set of unlabeled samples to calibrate the network. This makes PTQ more practical for real-world applications, especially when retraining is impractical due to resource constraints or data privacy concerns. Therefore, our focus in this paper is on designing an effective PTQ approach for medical imaging.

Although previous PTQ methods have achieved notable success in various scenarios, including convolutional neural networks (CNNs)^17^ and vision transformers (ViTs),^18^^–^^20^ a common limitation is their reliance on simulated quantization. In this approach, the quantization process is simulated during inference to approximate lower-precision computations, but the underlying model remains in high precision. As a result, there are no actual reductions in model size or meaningful improvements in real-world inference speed. This disconnect between simulated efficiency gains and practical performance limits the benefits of quantization in resource-constrained settings.

Moreover, recent advancements in deep learning, such as the 1.4B parameter STU-Net,^21^ highlight the transformative potential of scaling laws in medical image segmentation, demonstrating that larger models trained on appropriately large datasets achieve superior performance by capturing complex anatomical features. However, the computational and memory demands of such large-scale models present significant challenges for deployment in resource-constrained clinical environments. For models such as STU-Net, a real PTQ framework that ensures robustness to precision loss, enabling efficient real-time inference without compromising accuracy, remains in high demand. Such an approach would bridge the gap between the theoretical advantages of scaling laws and their practical utility, facilitating scalable and cost-effective deployment of high-capacity models in medical imaging.

**Fig. 1:**
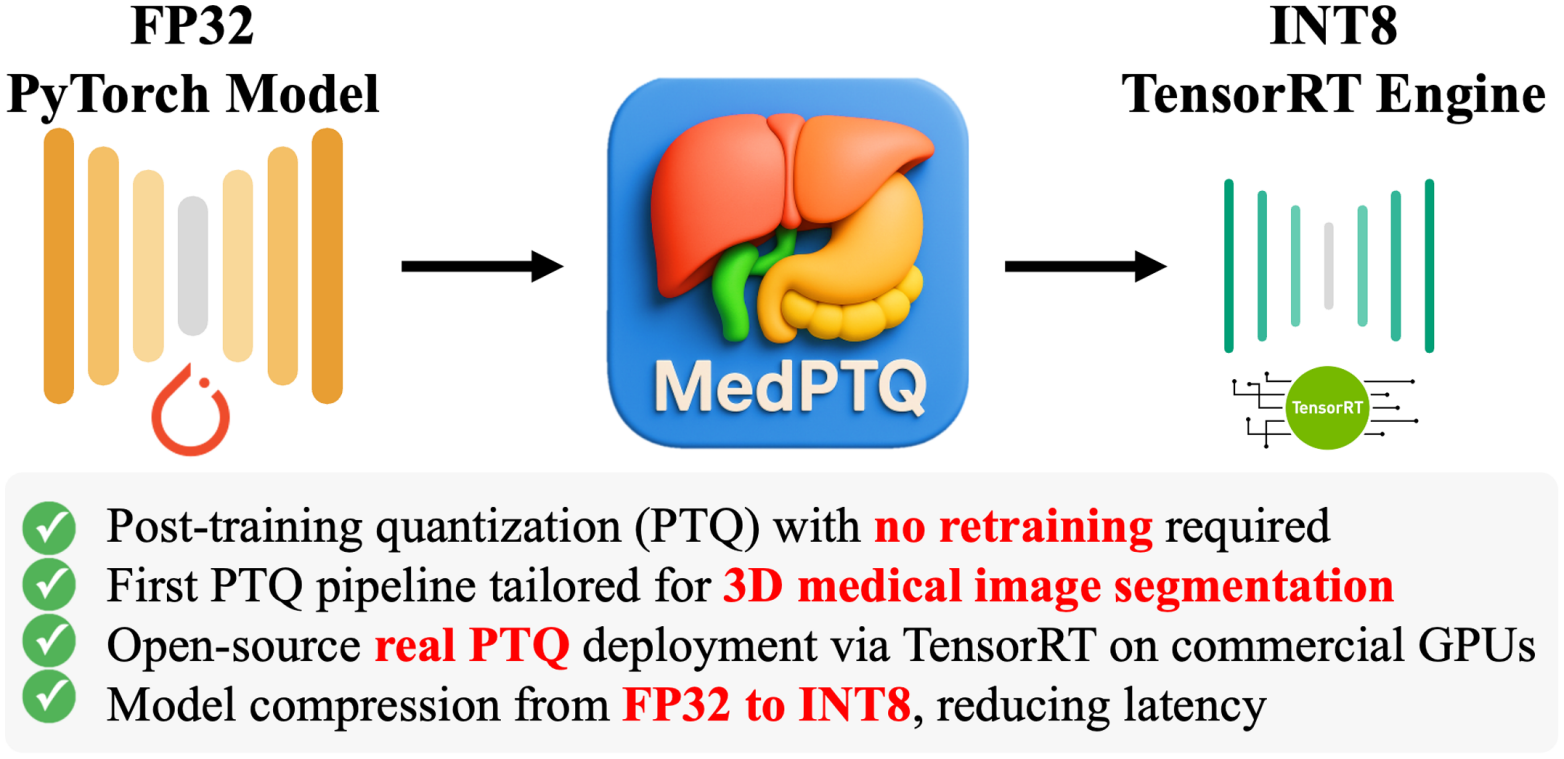
We introduce MedPTQ, an open-source pipeline for real post-training quantization that converts FP32 PyTorch models into INT8 TensorRT engines. By leveraging TensorRT for real INT8 deployment, MedPTQ reduces model size and inference latency while preserving segmentation accuracy for efficient GPU deployment.

**Fig. 2:**
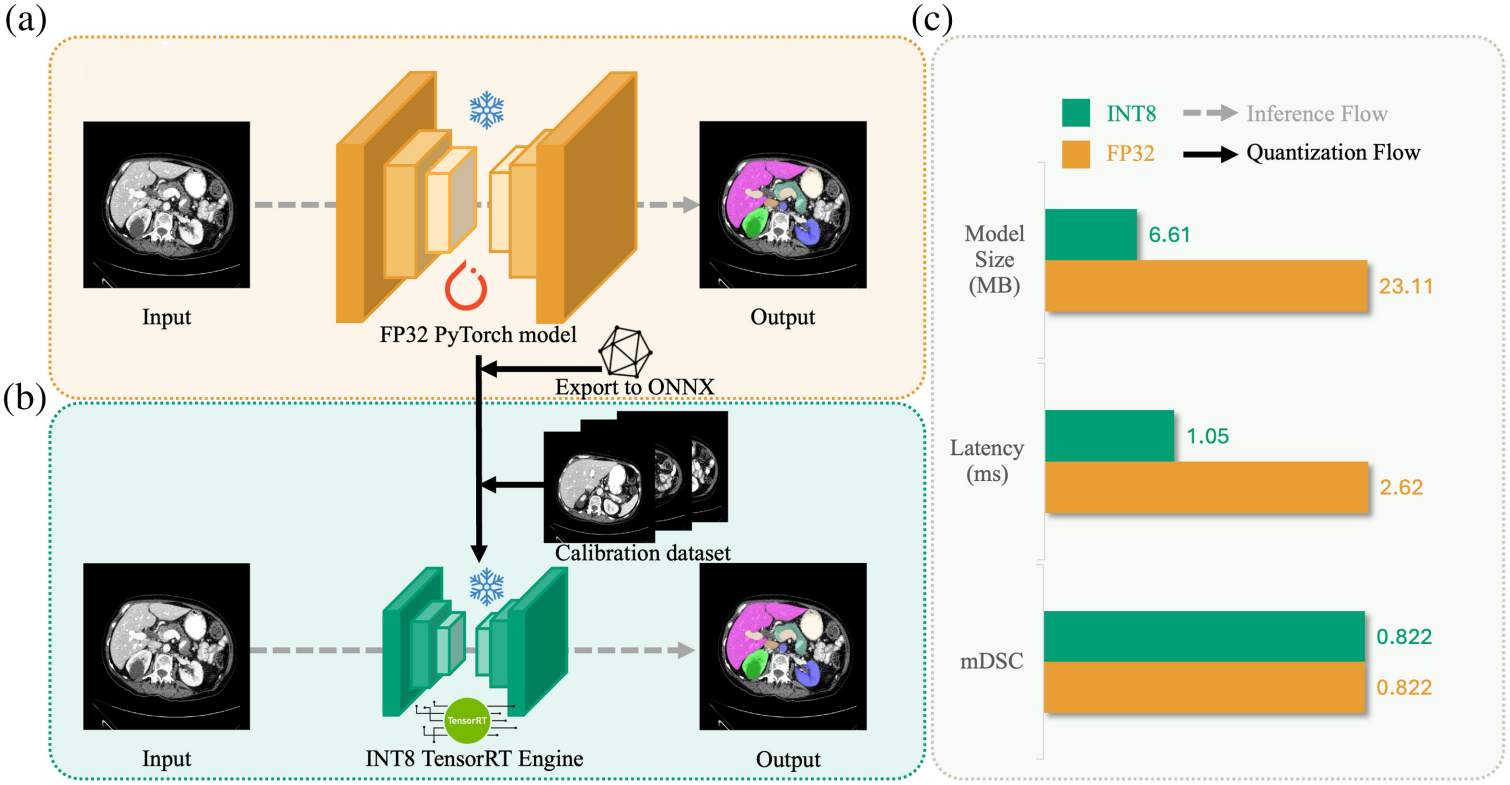
FP32 precision inference versus MedPTQ INT8 precision deployment. (a) FP32 precision inference. Previous 3D medical image segmentation commonly uses FP32 models, which results in larger model sizes, higher computational demands, and slower inference. (b) INT8 precision deployment. We propose MedPTQ, using TensorRT to convert FP32 models into INT8, enabling substantial reductions in model size and inference latency without compromising accuracy. (c) FP32 versus INT8 U-Net. As an example, U-Net’s model size decreases from 23.11 to 6.61 MB, and its inference latency drops from 2.62 to 1.05 ms while maintaining the same mDSC of 0.822.

To fulfill this need, we introduce MedPTQ, an open-source pipeline that implements real post-training quantization for 3D medical segmentation models. As illustrated in Fig. 1, MedPTQ converts FP32 models into optimized INT8 TensorRT engines, achieving substantial reductions in both model size and latency with no retraining. Our MedPTQ involves two key steps, as depicted in Figs. 2(a) and 2(b). First, starting from a pretrained FP32 PyTorch model (frozen for inference), we use NVIDIA TensorRT (NVIDIA TensorRT is a high-performance deep learning inference optimizer and runtime library that facilitates faster inference on NVIDIA GPUs through graph optimizations, precision calibration, and efficient memory management) to perform simulated quantization of both model weights and activations using an unlabeled calibration dataset. Second, the simulated quantized model is converted into a real INT8 TensorRT engine for deployment on real GPUs. TensorRT applies hardware-specific optimizations that enable efficient low-precision computations, resulting in tangible reductions in model size and faster inference times. As an example, Fig. 2(c) shows that for U-Net, the model size decreases from 23.11 to 6.61 MB (3.50× smaller) and inference latency drops from 2.62 to 1.05 ms (2.50× faster) while maintaining the same mean Dice similarity coefficient (mDSC) of 0.822.

**Fig. 3:**
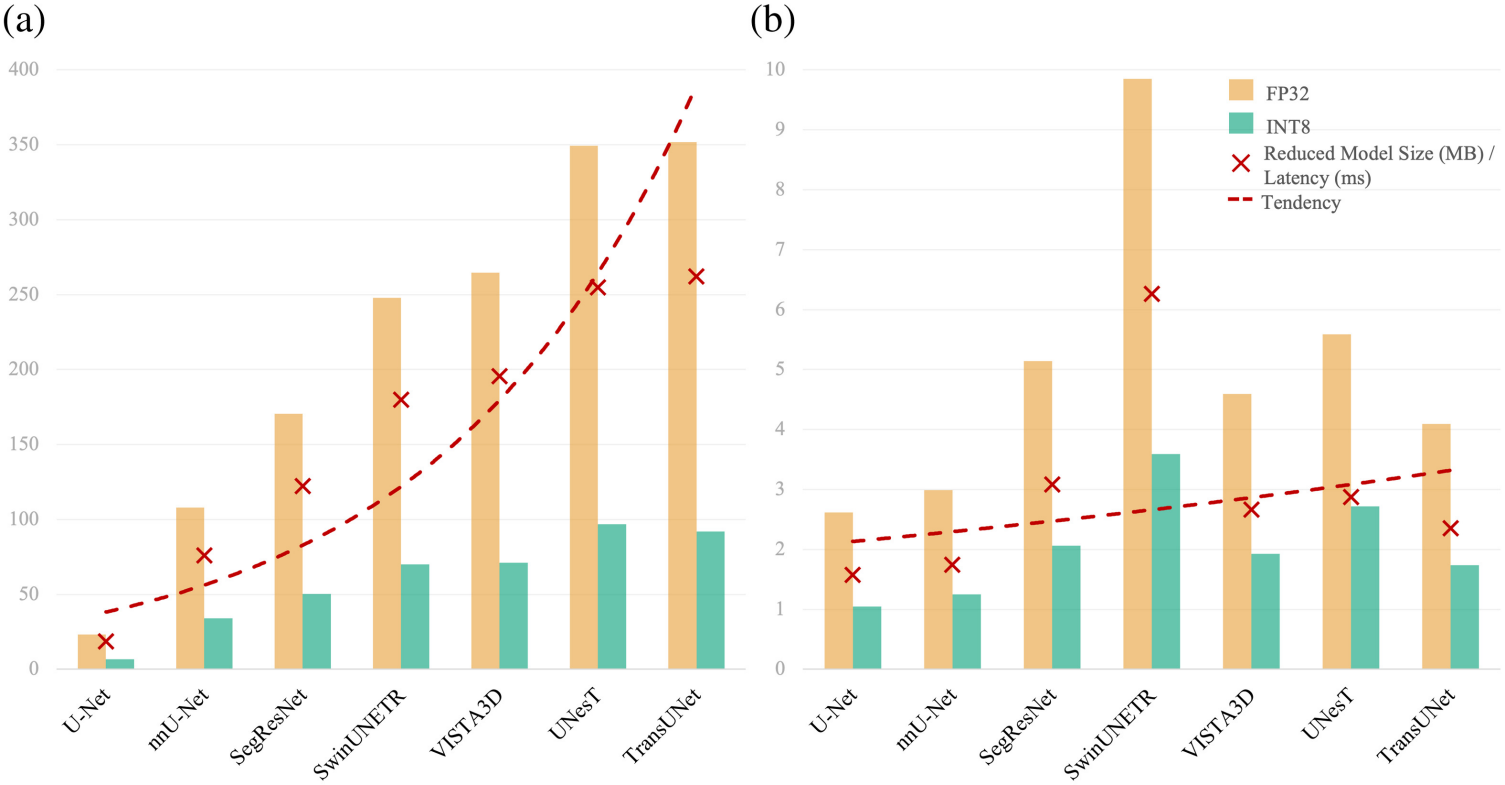
Scalability of INT8 quantization benefits. We analyze the relationship between model size and quantization efficiency across seven medical segmentation models. (a) As the FP32 model size (MB) increases, INT8 quantization yields progressively larger reductions in model size. The red x markers indicate the absolute model size reduction (FP32 − INT8, in MB) for each model, and the dashed trend line illustrates that larger models achieve greater compression. (b) Similar trend for inference latency (ms), where red x markers indicate the absolute latency reduction (FP32 − INT8, in ms) for each model. Overall, INT8 quantization provides increasing gains in both model size compression and inference latency as model complexity grows, highlighting its importance for scaling up 3D medical image segmentation.

To further demonstrate the effectiveness of MedPTQ, we benchmark seven state-of-the-art (SOTA) 3D medical segmentation models, i.e., U-Net,^22^ TransUNet,^23^ UNesT,^24^ nnU-Net,^5^ SwinUNETR,^25^ SegResNet,^26^ and VISTA3D,^27^ quantized to INT8 via our pipeline, against their FP32 counterparts. These evaluations span three datasets (TotalSegmentator V2,^28^ Whole Brain,^29^ and BTCV^30^), with results summarized in Figs. 3 and 5. Unlike simulated quantization, which only simulates INT8 computations but still relies on FP32 resources, MedPTQ consistently yields real reductions in model size and inference latency while preserving segmentation accuracy across representative models and datasets. In summary, the key contribution of this paper is threefold:

• Development of real post-training quantization in 3D medical image segmentation: We present MedPTQ, a pipeline that converts pretrained FP32 PyTorch models into INT8 TensorRT engines with no retraining using unlabeled calibration, delivering real reductions in model size and inference latency on modern GPUs while preserving segmentation accuracy.
• Disseminate the MedPTQ pipeline to the community as an open-source solution: We release MedPTQ as an open-source, reproducible codebase with calibration utilities, build/evaluation scripts, deployment examples, and pretrained checkpoints (U-Net, TransUNet, UNesT, nnU-Net, SwinUNETR, SegResNet, and VISTA3D), lowering the barrier to real INT8 deployment and enabling community adoption and extension.
• Benchmarking MedPTQ on various 3D medical segmentation models: We demonstrate MedPTQ’s robustness by successfully quantizing a broad set of SOTA 3D medical segmentation models, confirming the universal feasibility of real INT8 quantization. As both artificial intelligence (AI) model sizes and dataset sizes continue to grow in clinical practice, MedPTQ offers a crucial pathway toward resource-efficient, large-scale medical image analysis.

We note that MedPTQ leverages existing quantization infrastructure from NVIDIA TensorRT rather than proposing novel quantization algorithms. Our contribution lies in systematically demonstrating that real INT8 quantization is practically achievable for 3D medical segmentation while preserving diagnostic accuracy and providing a reproducible pipeline to facilitate community adoption.

## 2 Related Work

Model quantization has become a critical technique for deploying deep neural networks on resource-constrained hardware by reducing model size and computational demands. Quantization methods are generally categorized into two main approaches: quantization-aware training and post-training quantization.

Quantization-aware training integrates quantization into the training process, allowing the model to learn and adapt to quantization effects during training. By incorporating discrete constraints directly into the backpropagation algorithm,^31^ QAT methods enable networks to maintain high accuracy even at low bit-widths. To facilitate gradient propagation through quantized variables, many QAT methods including XNOR-Net,^32^ QIL,^33^ 3DQ,^34^ and MedQ^35^ utilize the straight-through estimator,^36^ which approximates the gradient of the non-differentiable quantization function. Some QAT approaches utilize differentiable approximations of the quantization function during training, which are then replaced with hard quantization during inference. DoReFa-Net^37^ quantizes weights, activations, and even gradients to low bit-widths using differentiable quantizers. Differentiable soft quantization (DSQ)^38^ introduces a differentiable function that smoothly approximates the quantizer. Although QAT methods can achieve high accuracy, they require integration into the training pipeline from the beginning. In practice, many state-of-the-art medical segmentation models are released as FP32 pretrained checkpoints without QAT support. Applying QAT to these existing models would require retraining with access to the original training code and datasets, which may not always be available due to data privacy concerns^39^^–^^41^ or the substantial size of medical imaging datasets.^42^^–^^44^ PTQ addresses this limitation by quantizing pretrained models directly, using only a small set of unlabeled samples for calibration.

Post-training quantization method quantizes a pre-trained model without additional training, using only a small set of unlabeled data for calibration. This makes PTQ more practical for real-world applications, especially when retraining is impractical due to resource constraints or data privacy concerns inherent in medical imaging. In the medical imaging domain, two main architectures are prevalent: CNNs and transformers. Numerous successful works have introduced quantization methods specifically designed for these architectures. For CNNs, AdaRound^45^ formulates quantization as an optimization problem by introducing a learnable rounding mechanism for weights. BRECQ^46^ extends AdaRound by implementing block-wise reconstruction, optimizing quantization parameters across blocks of layers to capture inter-layer dependencies. QDrop^47^ incorporates a dropout mechanism during the reconstruction process, adding randomness to enhance the robustness and flatness of the optimized model. PD-Quant^48^ leverages a prediction difference metric to optimize network blocks globally, introducing global information into the quantization parameter optimization. When quantizing transformers, previous works have focused on solving post-softmax distribution problems and addressing activation distribution outliers in intermediate layers. FQ-ViT^49^ simulates the non-uniform distribution of attention maps by employing a log2 quantizer and proposes an integer approximation of the exponential function for softmax quantization, also introducing power-of-two quantization for the layer normalization layer. PTQ4ViT^50^ introduces twin uniform quantization to handle post-softmax and post-GELU activation distributions. RepQ-ViT^51^ proposes $\log\;\sqrt{2}$ to better adapt to the distribution of post-softmax results. PTQ4SAM^52^ proposes a bimodal integration strategy, applying a mathematically equivalent sign operation to transform the bimodal distribution of key linear output activations into a more easily quantized normal distribution. In addition, it introduces adaptive granularity quantization for softmax by searching for the optimal power-of-two base to address substantial variations in post-softmax distributions. Although these previous PTQ methods effectively address the quantization challenges in CNN and transformer architectures, they primarily rely on simulated quantization techniques that do not result in actual reductions in model size, computational demand, or inference latency. In this paper, we introduce MedPTQ that performs real quantization on modern GPUs, optimizing the inference process and achieving remarkable resource savings. Although we use the term simulated quantization to highlight the lack of real-world latency or memory benefits, we acknowledge that these PTQ methods contribute important techniques (e.g., handling activation outliers, improved rounding, and calibration strategies) that remain valuable for advancing quantization research in both CNNs and transformers.

ONNX^53^ and TensorRT:^54^ To enable interoperability across deep learning frameworks, the open neural network exchange (ONNX) provides a standardized representation of models that can be exported from PyTorch or TensorFlow and subsequently optimized by deployment engines. Among these engines, NVIDIA TensorRT is widely adopted for high-performance inference on GPUs. TensorRT parses ONNX models, applies graph optimizations such as operator fusion and constant folding, and generates hardware-specific kernels (including INT8 and FP16). This integration of ONNX and TensorRT has made it possible to bridge research prototypes and practical deployment, providing a natural foundation for MedPTQ.

Although these prior PTQ methods contribute important techniques for mitigating accuracy degradation, such as learnable rounding and block-wise reconstruction, they are primarily algorithm-oriented and focus on improving quantization accuracy under simulated conditions. In contrast, MedPTQ is deployment-oriented, with the primary goal of achieving real-world efficiency gains on modern GPUs. Our experiments demonstrate that for 3D medical image segmentation models, standard TensorRT calibration with entropy-based scale estimation is sufficient to preserve segmentation accuracy (mDSC degradation ≤0.006) while delivering tangible reductions in model size and inference latency. Nevertheless, these advanced quantization techniques remain valuable and could be integrated into our framework if future models or lower-bit quantization (e.g., INT4) exhibit greater sensitivity to quantization errors.

## 3 Method

**Fig. 4:**
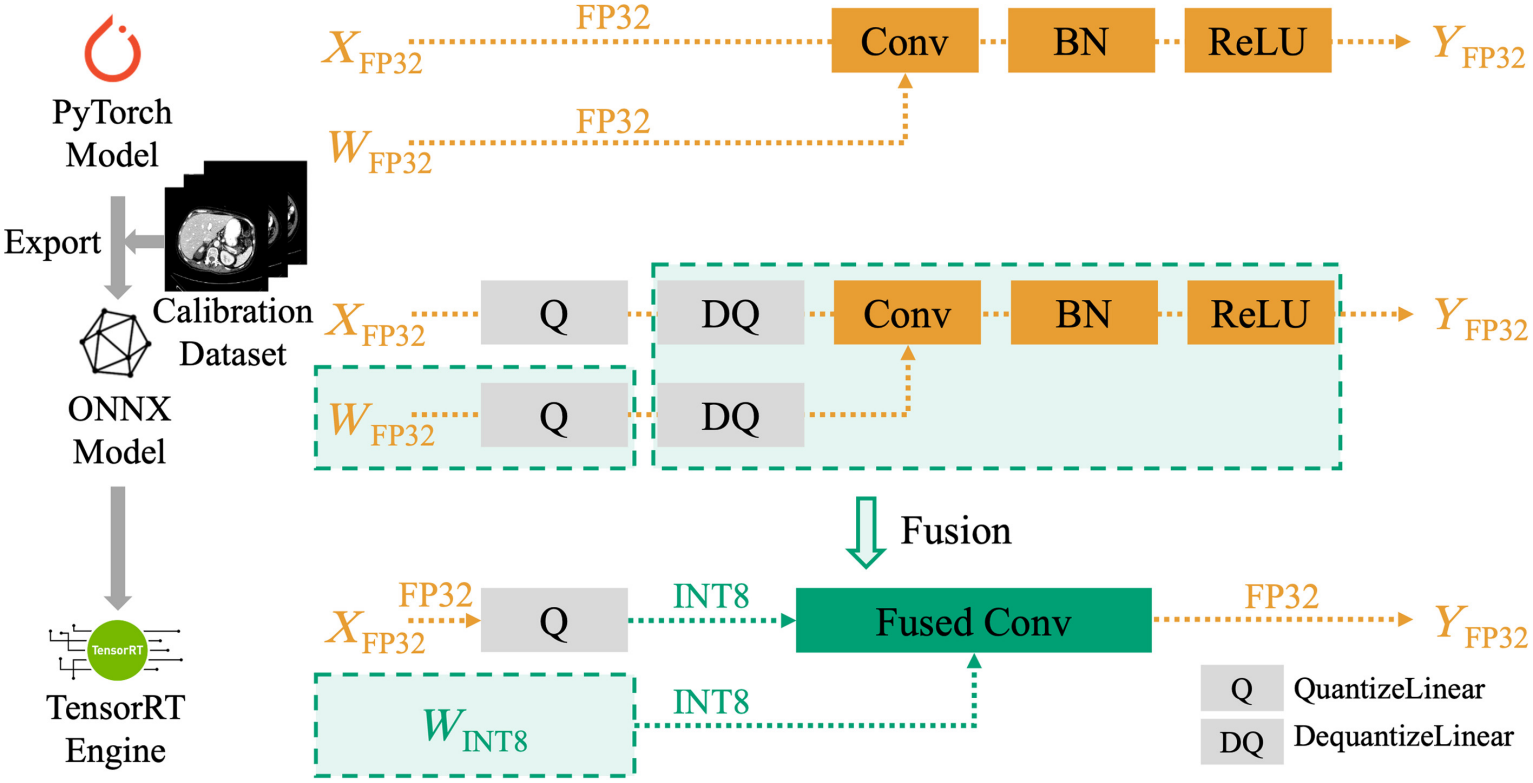
Overview of MedPTQ. The top row illustrates the original FP32 pipeline, where both activation X and weight W are in full precision and passed through Conv-BN-ReLU sequentially. The middle row shows the simulated quantization stage (Sec. 3.1): QuantizeLinear and De QuantizeLinear nodes are inserted after both activations and weights to simulate INT8 quantization semantics, but the model still executes in FP32. The bottom row demonstrates the real INT8 TensorRT engine (Sec. 3.2), where TensorRT fuses the FP32 weights with their associated QuantizeLinear into INT8 weights, and merges the activation DequantizeLinear, weight DequantizeLinear, convolution, BN, and ReLU into a single fused convolution block. This fusion enables the use of optimized INT8 convolution kernels, thereby reducing memory traffic and improving efficiency while preserving accuracy.

Overview: In this section, we present our proposed MedPTQ designed to implement real INT8 quantization on SOTA medical segmentation models on modern GPUs, as shown in Fig. 4. Our method aims to reduce model size and inference latency without retraining, thus making advanced models more accessible in resource-constrained environments. MedPTQ consists of two main components: first, we leverage NVIDIA TensorRT to perform simulated quantization by inserting QuantizeLinear and DequantizeLinear nodes into the open neural network exchange (ONNX) model with an unlabeled calibration dataset (Sec. 3.1), simulating the quantization process while still relying on FP32 resources. Second, we convert this simulated quantized model into a real INT8 TensorRT engine for efficient deployment on modern GPUs (Sec. 3.2). During this conversion, TensorRT detects the QuantizeLinear and DequantizeLinear nodes to perform real INT8 quantization. Our MedPTQ addresses the limitations of previous PTQ methods that rely on simulated quantization, which simulates lower-precision computations without yielding actual reductions in model size or improvements in inference speed. By converting simulated quantization into real quantization, the resulting TensorRT engine stores weights in INT8 format and executes computations using INT8 kernels, enabling actual reductions in model size and inference latency on modern GPUs. We focus on a practical real-INT8 quantization for 3D segmentation that includes calibration, explicit Q/DQ insertion, and TensorRT fusion, rather than proposing a new quantizer.

### 3.1 Perform Simulated Quantization on ONNX

In the first component of MedPTQ, we perform simulated quantization on both the weights and activations of a pre-trained network using an unlabeled calibration dataset. To enable this process, we first export the pre-trained PyTorch model into the ONNX format

$$\text{PyTorch}\;\text{Model}\overset{\text{Export}}{\to }\mathrm{ONNX}\;\text{Model}.$$

ONNX is an open standard that provides a common model format across frameworks. This conversion gives us a portable representation that can be processed by different optimization tools, including TensorRT.

#### 3.1.1 Simulation of INT8 quantization with ModelOpt

After conversion, we use the NVIDIA TensorRT Model Optimizer (ModelOpt) to insert QuantizeLinear and DequantizeLinear nodes after both activations and weights

$$\mathrm{ONNX}\;\text{Model}\overset{\text{ModelOpt}}{\to }\text{Simulated Quantized Model}.$$

Here, QuantizeLinear maps FP32 values to simulated INT8 integers based on scale factors and zero-points obtained from calibration, whereas DequantizeLinear converts them back into FP32 so that the model can still run with floating-point kernels. Formally, for a floating-point value x, the quantized integer $x_{q}$ is computed as

$$x_{q}=\text{clamp}(\lfloor \frac{x}{s}\rceil +z,0,2^{k}-1),$$ (1)

where $s=\frac{x_{\max}-x_{\min}}{2^{k}-1}$ is the scale factor, $z=-\lfloor x_{\min}/s\rceil$ is the zero-point, k is the bit-width, and clamp(·,a,b) restricts the values to the integer range. This procedure preserves the numerical semantics of INT8 operations while still executing on FP32 hardware. Note that this simulated quantization stage does not provide computational benefits; it serves only to determine calibration parameters for the subsequent real quantization step (Sec. 3.2), where true INT8 computation yields actual speedup.

### 3.2 Converting to Real Quantization Using NVIDIA TensorRT

In the second step of MedPTQ, we transform the simulated quantized ONNX model into a real INT8 engine optimized for deployment using NVIDIA TensorRT. This step replaces the simulated Q/DQ operations with true INT8 computation on GPUs.

#### 3.2.1 Building the TensorRT engine

We input the simulated quantized ONNX model into TensorRT, which parses the graph and performs operator fusion to create efficient INT8 kernels

$$\text{Simulated Quantized Model}\overset{\text{TensorRT}}{\to }\mathrm{INT}8\;\text{Engine}.$$

During this process, TensorRT fuses the FP32 weights with their associated QuantizeLinear into INT8 weights, so that they are stored and accessed directly in 8-bit form. At the same time, the activation DequantizeLinear, weight DequantizeLinear, convolution, batch normalization, and ReLU layers are merged into a single fused convolution block. This block is then executed by optimized INT8 convolution kernels. The element-wise multiplications are performed in INT8 precision using dedicated tensor cores, whereas the products are accumulated in INT32 to prevent overflow. This accumulation strategy is standard practice in GPU implementations and preserves numerical stability without sacrificing the throughput benefits of INT8 computation. The fused kernel then applies the required scaling, bias, and activation in one pass.

By converting simulated quantization into real quantization in this way, the resulting TensorRT engine avoids unnecessary data conversions, reduces memory traffic, and fully utilizes GPU INT8 hardware. As a result, we achieve significant reductions in model size and inference latency while maintaining the same segmentation accuracy as FP32 models.

## 4 Experiments and Results

### 4.1 Dataset

BTCV^30^ consists of 70 CT volumes with 13 labeled anatomies. They are randomly selected from a combination of an ongoing colorectal cancer chemotherapy trial and a retrospective ventral hernia study. Of these, 50 CT volumes, which are publicly available through the MICCAI 2015 Multi-Atlas Labeling Challenge, are used to pre-train our U-Net and TransUNet models. The remaining 20 CT volumes are used for evaluation.

TotalSegmentator V2^28^ includes 1228 full-body CT volumes with 117 labeled anatomies, created by the Department of Research and Analysis at University Hospital Basel. We use 200 of these CT volumes to evaluate nnU-Net, SegResNet, SwinUNETR, and VISTA3D across 104 labels. The remaining 1028 volumes are used to pre-train these models.

Whole Brain Segmentation Dataset^29^ combines 4859 T1-weighted (T1w) MRI volumes collected from eight different sites, with segmentation labels generated by a multi-atlas segmentation pipeline. Among these volumes, 50 come from the Open Access Series on Imaging Studies (OASIS) dataset^55^ and have been manually traced to 133 labels based on the BrainCOLOR protocol^56^ by Neuromorphometrics Inc. We use these 50 manually labeled scans to evaluate our UNesT models, whereas the remaining 4809 scans are used for pre-training.

### 4.2 Implementation Details

U-Net and TransUNet are pre-trained and evaluated on the BTCV dataset, UNesT is pre-trained and evaluated on the Whole Brain Segmentation dataset, nnU-Net, SegResNet, SwinUNETR, and VISTA3D are pre-trained and evaluated on TotalSegmentator V2. Except for nnU-Net, which follows its default training plan and original learning rate, these models share the same data augmentation and pre-processing steps and are trained on a single NVIDIA RTX 4090 GPU with an input volume size of 96×96×96. They employ the Adam optimizer starting at 1e−4 and a weight decay of 1e−5, with the learning rate dynamically adjusted based on the combined Dice and cross-entropy (DiceCE) loss. After training, all models are quantized into INT8 engines using NVIDIA TensorRT, retaining FP32 for input and output, and both quantization and evaluation are performed on a single NVIDIA RTX 4090 GPU. Segmentation accuracy is measured via the mDSC, and to compare model efficiency between INT8 and FP32 versions, we monitor model size, GPU memory usage during inference, and inference latency.

### 4.3 Quantization Results of Segmentation Models

**Fig. 5:**
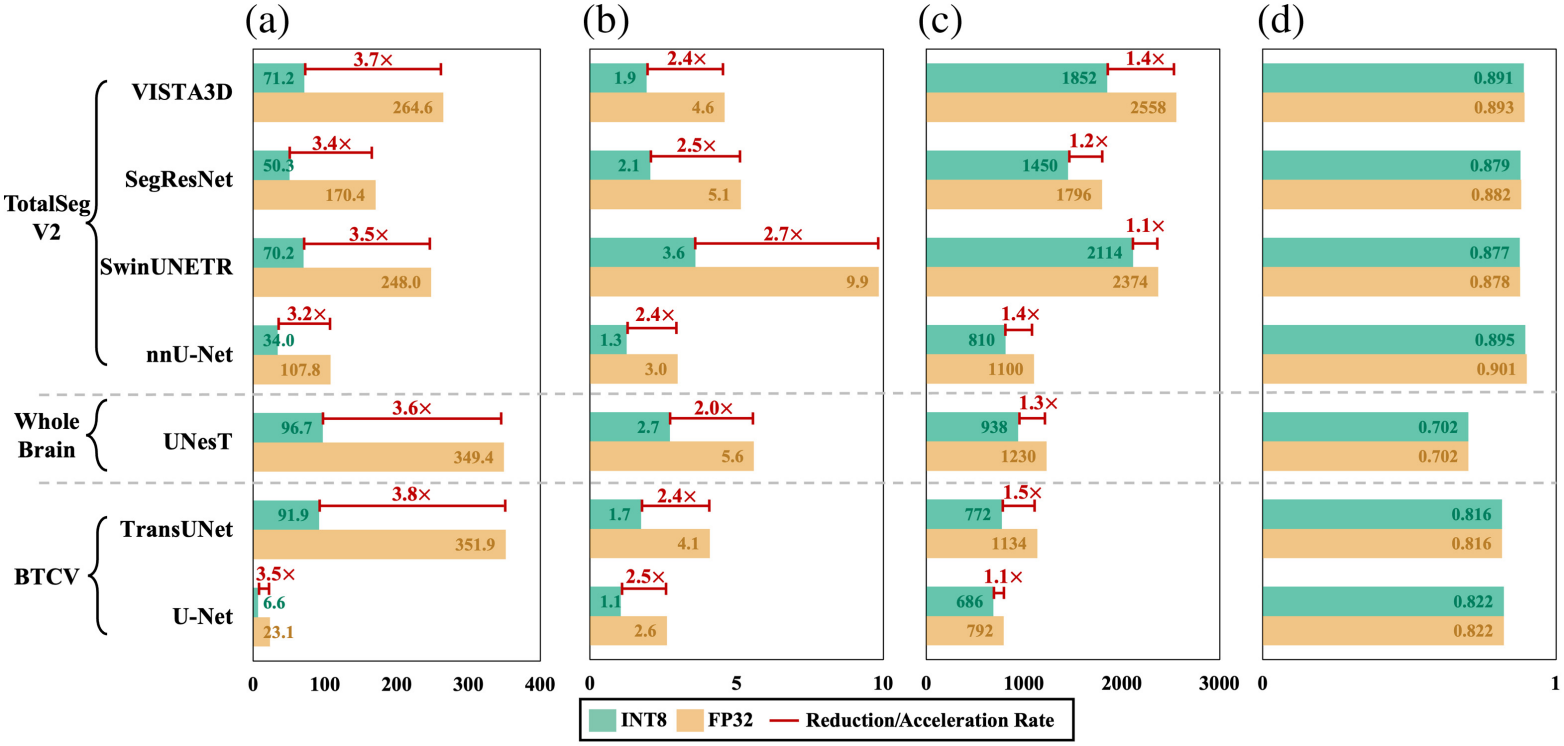
Quantization results of MedPTQ across seven medical segmentation models. We evaluate MedPTQ on seven models across three datasets: TotalSegmentator V2 (VISTA3D, SegResNet, SwinUNETR, nnU-Net), Whole Brain (UNesT), and BTCV (TransUNet, U-Net). Four metrics are reported. (a) Model size (MB). (b) Inference latency (ms). (c) GPU memory usage (MB). (d) mDSC. Compared with FP32 counterparts, INT8 models achieve 3.2× to 3.8× model size reduction, 2.0× to 2.7× latency speedup, and 1.1× to 1.5× GPU memory reduction while maintaining virtually unchanged segmentation accuracy (|ΔmDSC|≤0.006).

We evaluate MedPTQ using seven SOTA medical segmentation models, i.e., U-Net, TransUNet, UNesT, nnU-Net, SwinUNETR, SegResNet, and VISTA3D, across three datasets with different numbers of samples (N) and number of classes (C), i.e., BTCV (N=20, C=13), Whole Brain Segmentation (N=50, C=133), and TotalSegmentator V2 (N=200, C=104). To eliminate inconsistencies among libraries (PyTorch versus TensorRT), all models are converted to TensorRT engines for both FP32 and INT8. As shown in Fig. 5, the INT8 quantized models achieve reductions in model size ranging from 3.2× to 3.8× and reductions in inference latency from 2.0× to 2.7× while maintaining nearly identical mDSC performance compared with their FP32 counterparts (absolute ΔmDSC≤0.006). These results demonstrate that our MedPTQ delivers real-world gains in efficiency, especially beneficial when working with large-scale datasets that typically require substantial inference time. We also measure GPU memory usage during inference to evaluate the impact of quantization on feature map memory. As shown in Fig. 5(c), INT8 quantization reduces GPU memory consumption by 1.1× to 1.5× across all models. This reduction is notably smaller than the model size compression ratio (3.2× to 3.8×), confirming that feature map memory is the dominant factor in runtime GPU usage. This discrepancy arises because TensorRT optimizes memory usage by reusing buffers across layers rather than allocating memory for all feature maps simultaneously. As a result, runtime GPU memory reflects peak activation memory plus workspace allocations, rather than the sum of all layer activations. Although INT8 quantization compresses both weights and activations, the workspace memory required by convolution algorithms remains largely unaffected by numerical precision. Nevertheless, the memory savings combined with the substantial latency reduction make MedPTQ valuable for deployment scenarios where both speed and memory are constrained.

### 4.4 Impact of Quantization on Small Structures

Although mDSC provides a useful summary metric, it can be dominated by large anatomical structures and may obscure potential quantization-induced degradation in smaller, clinically important organs. To address this concern, we conduct a detailed class-level analysis on the BTCV dataset, which contains organs of varying sizes including several small structures that are often clinically significant but challenging to segment accurately.

Table 1 reports mDSC and mean normalized surface distance (mNSD) for each of the 13 anatomical classes in the BTCV dataset. We highlight five small organs: gallbladder, esophagus, pancreas, right adrenal gland, and left adrenal gland. These structures occupy relatively few voxels in the CT volumes and are therefore more susceptible to quantization errors, as small numerical perturbations can have a proportionally larger impact on boundary delineation.

**Table 1:** Per-class segmentation results on the BTCV dataset. We report mDSC and mNSD for both FP32 and INT8 models. Results for small organs (gallbladder, esophagus, pancreas, and adrenal glands) are in bold. Results show that INT8 quantization has minimal impact across all classes, including small structures.

Class	U-Net				TransUNet			
	mDSC		mNSD		mDSC		mNSD	
	Original (FP32)	MedPTQ (INT8)	Original (FP32)	MedPTQ (INT8)	Original (FP32)	MedPTQ (INT8)	Original (FP32)	MedPTQ (INT8)
Spleen	0.956	0.956	0.740	0.740	0.951	0.951	0.725	0.725
Right kidney	0.897	0.896	0.712	0.713	0.894	0.894	0.691	0.690
Left kidney	0.889	0.889	0.700	0.701	0.877	0.878	0.682	0.683
Gallbladder	**0.530**	**0.527**	**0.610**	**0.561**	**0.533**	**0.533**	**0.564**	**0.562**
Esophagus	**0.793**	**0.793**	**0.599**	**0.598**	**0.789**	**0.788**	**0.564**	**0.563**
Liver	0.967	0.967	0.665	0.666	0.966	0.966	0.661	0.660
Stomach	0.889	0.887	0.514	0.510	0.893	0.893	0.502	0.500
Aorta	0.901	0.901	0.710	0.710	0.899	0.899	0.695	0.693
Inferior vena cava	0.847	0.847	0.588	0.587	0.851	0.850	0.587	0.586
Portal and splenic vein	0.776	0.776	0.590	0.591	0.757	0.757	0.562	0.564
Pancreas	**0.812**	**0.812**	**0.530**	**0.530**	**0.794**	**0.794**	**0.508**	**0.508**
Right adrenal gland	**0.740**	**0.740**	**0.673**	**0.672**	**0.702**	**0.703**	**0.640**	**0.641**
Left adrenal gland	**0.693**	**0.696**	**0.613**	**0.615**	**0.707**	**0.708**	**0.616**	**0.618**
Mean	0.822	0.822	0.634	0.630	0.816	0.816	0.615	0.615

Our results demonstrate that INT8 quantization via MedPTQ preserves segmentation accuracy across all organ classes, including small structures. For U-Net, the maximum class-level DSC change is 0.003 (gallbladder: 0.530 → 0.527 and left adrenal gland: 0.693 → 0.696), whereas TransUNet shows even smaller variations with a maximum class-level DSC change of 0.001. Importantly, the small organs exhibit comparable stability to large organs under quantization, with no evidence of disproportionate accuracy loss.

These findings suggest that the precision reduction from FP32 to INT8 does not introduce systematic errors that preferentially affect small or thin anatomical structures. This is clinically relevant because organs such as the pancreas and adrenal glands, despite their small size, play critical roles in diagnosing conditions ranging from pancreatitis to adrenal tumors. The robustness of MedPTQ across organ sizes further validates its applicability for deployment in clinical workflows where accurate segmentation of both large and small structures is essential.

### 4.5 Scaling Analysis

To evaluate the impact of scaling effectiveness on TotalSegmentator V2 (N=200, C=104), we compare the performance and efficiency of STU-Net-S (14M parameters) and STU-Net-H (1.4B parameters).^21^ As shown in Table 2, by scaling the model size, segmentation accuracy (measured by mDSC) increases from 0.837 to 0.869, demonstrating the benefits of scaling for capturing complex anatomical structures.

**Table 2:** Quantization results of STU-Net on TotalSegmentator V2. We evaluate the impact of scaling effectiveness using STU-Net (scaling up from 14M to 1.4B parameters) on TotalSegmentator V2 (N=200, C=104). The segmentation performance (measured by mDSC) increases from 0.837 to 0.869, demonstrating the benefits of scaling for capturing complex anatomical structures. Then, by applying MedPTQ, the INT8-quantized STU-Net-H shrinks by 3.65× (5559.40/1519.78) and reduces inference latency by 3.26× (98.45/30.15) while preserving competitive segmentation performance as its FP32 counterpart. The results indicate that MedPTQ effectively addresses the computational challenges of scaling.

Dataset	AI models	Model size (MB)		Latency (ms)		mDSC	
		Original (FP32)	MedPTQ (INT8)	Original (FP32)	MedPTQ (INT8)	Original (FP32)	MedPTQ (INT8)
TotalSeg V2^28^	STU-Net-S^21^	55.70	20.52	2.59	1.02	0.837	0.835
(N=200, C=104)	STU-Net-H	5559.40	1519.78	98.45	30.15	0.869	0.866

N, number of samples; C, number of classes.

Despite the performance gains, scaling introduces higher computational demands. On TotalSegmentator V2, inference latency for STU-Net increases from 2.59 to 98.45 ms, making it prohibitively slow when processing large-scale datasets. After applying MedPTQ, the INT8 quantized STU-Net-H model size decreases by 3.65× (5559.40/1519.78), and its inference latency drops by 3.26× (98.45/30.15) while maintaining comparable mDSC scores to the FP32 counterpart. Compared with smaller STU-Net-S that already achieve a latency of under 10 ms, our PTQ approach yields even more pronounced gains for large-scale models such as STU-Net-H, cutting latency by an additional 68.3 ms. Furthermore, the INT8 quantized STU-Net-H achieves a higher compression ratio in terms of model size (3.65×) than STU-Net-S (2.71×), because smaller models are more impacted by overheads and non-quantizable layers, whereas larger models have a greater proportion of parameters that benefit from quantization. Consequently, MedPTQ proves particularly beneficial for large-scale models, making it a powerful solution for deploying resource-efficient deep learning systems.

These results demonstrate that MedPTQ effectively addresses the computational challenges of scaling, enabling the deployment of large-scale models such as STU-Net in resource-constrained environments. This highlights the synergy between scaling laws and quantization for advancing medical image segmentation.

## 5 Discussion

The results of our experiments demonstrate that our proposed MedPTQ effectively reduces model size, computational demands, and inference latency without compromising model performance. Notably, the quantized INT8 models maintain segmentation accuracy comparable to their FP32 counterparts, as indicated by the nearly unchanged mDSC. This finding is noteworthy because it challenges the common concern that quantization, particularly at low bit-widths such as INT8, inherently leads to a degradation in model performance.

### 5.1 Robustness Across Models and Datasets

The effectiveness of MedPTQ across SOTA medical segmentation models (U-Net, TransUNet, UNesT, nnU-Net, SwinUNETR, SegResNet, and VISTA3D) and across datasets (BTCV, Whole Brain Segmentation dataset, and TotalSegmentator V2) underscores its generalizability. These models vary in architecture and complexity, and the datasets cover a range of anatomical structures and imaging modalities. The consistent performance across these variations indicates that MedPTQ is robust and adaptable to various AI models used in the medical imaging domain.

### 5.2 GPU Memory and Quantization

MedPTQ performs INT8 quantization on both weights and activations (W8A8). As shown in Fig. 5(c), the GPU memory reduction (1.1× to 1.5×) is notably smaller than model size compression (3.2× to 3.8×), confirming that feature map memory dominates runtime GPU usage. This discrepancy arises because TensorRT optimizes memory usage by reusing buffers across layers rather than allocating memory for all feature maps simultaneously. As a result, runtime GPU memory reflects peak activation memory plus workspace allocations, rather than the sum of all layer activations. Although INT8 quantization compresses both weights and activations, the workspace memory required by convolution algorithms remains largely unaffected by numerical precision. Therefore, although MedPTQ reduces GPU memory usage, the reduction is modest, suggesting that INT8 quantization alone may not enable deployment on significantly smaller GPUs. Achieving greater memory savings would require additional techniques, which we leave for future work.

### 5.3 Clinical Applications

Although training 3D segmentation models is computationally intensive, it is typically performed once offline. In clinical practice, inference is performed repeatedly on every new patient scan. For large-scale applications such as population screening or retrospective cohort analysis involving thousands of volumes, inference latency becomes a critical bottleneck. In addition, many clinical environments operate with limited computational resources, where efficient inference enables the deployment of advanced AI models that would otherwise be impractical. Diagnostic accuracy is also critical in the medical domain, as it directly affects patient care and treatment decisions. Maintaining model performance after quantization is essential to ensure that efficiency gains do not compromise clinical use. MedPTQ maintains mDSC on high-resolution segmentation tasks and can be integrated into clinical workflows. At the same time, overall mDSC does not fully capture clinical risk; subtle, diagnostically important features should be verified in targeted evaluations.

### 5.4 Potential Limitations and Future Work

Despite the promising results, there are certain limitations to MedPTQ that need to be addressed. One notable limitation stems from the use of NVIDIA TensorRT for converting simulated quantization into real INT8 computations. TensorRT may not fully support models with dynamic blocks or layers that require runtime flexibility, such as those involving variable input sizes or conditional operations. These dynamic architectures can pose compatibility issues with TensorRT’s optimization and quantization processes, potentially limiting the applicability of MedPTQ to a subset of AI models. Addressing this limitation would involve enhancing the compatibility of TensorRT with dynamic model components or exploring alternative optimization tools that can handle such architectures effectively.

Future work will include more clinical validation (e.g., edge case audits and radiologist review) and calibration sensitivity studies. A further direction is quantization to even lower precision, such as a 4-bit integer (INT4). Exploring INT4 quantization has the potential to further reduce model size and computational requirements, offering additional benefits for deployment in extremely resource-constrained environments. However, achieving INT4 quantization without compromising model accuracy presents substantial challenges. The reduced precision can introduce considerable quantization errors, leading to performance degradation. Developing advanced quantization techniques, such as adaptive quantization strategies or error compensation methods, will be crucial to maintain model performance at these lower precisions. In addition, researching hardware accelerators optimized for INT4 computations could enhance the practical feasibility of deploying such quantized models in real-world medical imaging applications.

## 6 Conclusion

We have introduced MedPTQ, a real post-training quantization pipeline that delivers INT8 inference for SOTA 3D AI models in medical imaging segmentation. MedPTQ effectively reduces real-world model size, computational requirements, and inference latency without compromising segmentation accuracy on modern GPUs, as evidenced by mDSC comparable to full-precision baselines. We validate MedPTQ across a diverse set of AI architectures, ranging from CNN-based to transformer-based models, and a wide variety of medical imaging datasets. These datasets are collected from multiple hospitals with distinct imaging protocols, cover different body regions (such as brain, abdomen, or full body), and include multiple imaging modalities (CT and MRI). Collectively, these results highlight our MedPTQ’s strong generalizability and adaptability for a broad spectrum of medical imaging tasks.

By preserving diagnostic accuracy while enhancing computational efficiency, MedPTQ holds considerable potential for clinical integration. It enables the deployment of advanced AI models in resource-constrained environments, facilitating faster and more efficient diagnostics without compromising patient care.

## Data Availability

The code is available on GitHub (https://github.com/hrlblab/PTQ). All datasets are publicly available.
